# A Conserved Virulence Plasmidic Region Contributes to the Virulence of the Multiresistant *Escherichia coli* Meningitis Strain S286 Belonging to Phylogenetic Group C

**DOI:** 10.1371/journal.pone.0074423

**Published:** 2013-09-26

**Authors:** Chloé Lemaître, Farah Mahjoub-Messai, Damien Dupont, Valérie Caro, Laure Diancourt, Edouard Bingen, Philippe Bidet, Stéphane Bonacorsi

**Affiliations:** 1 Equipe d'ccueil EA3105, Univ Paris Diderot, Sorbonne Paris Cité, Paris, France; 2 Laboratoire de Microbiologie, AP-HP, Hôpital Robert Debré, Paris, France; 3 Genotyping of Pathogens and Public Health, Institut Pasteur, Paris, France; University of Münster, Germany

## Abstract

Recent isolation of the non-K1 *Escherichia coli* neonatal meningitis strain S286, belonging to phylogroup C, which is closely related to major group B1, and producing an extended-spectrum beta-lactamase, encouraged us to seek the genetic determinants responsible for its virulence. We show that S286 belongs to the sequence O type ST23_O78_ and harbors 4 large plasmids. The largest one, pS286colV (∼120 kb), not related to resistance, contains genes characteristic of a Conserved Virulence Plasmidic (CVP) region initially identified in B2 extra-intestinal avian pathogenic *E. coli* (APEC) strains and in the B2 neonatal meningitis *E. coli* strain S88. The sequence of this CVP region has a strong homology (98%) with that of the recently sequenced plasmid pChi7122-1 of the O78 APEC strain Chi7122. A CVP plasmid-cured variant of S286 was less virulent than the wild type strain in a neonatal rat sepsis model with a significant lower level of bacteremia at 24 h (4.1±1.41 versus 2.60±0.16 log CFU/ml, p = 0.001) and mortality. However, the mortality in the model of adult mice was comparable between wild type and variant indicating that pS286colV is not sufficient by itself to fully explain the virulence of S286. Gene expression analysis of pS286colV in iron depleted environment was very close to that of pS88, suggesting that genes of CVP region may be expressed similarly in two very different genetic backgrounds (group C versus group B2). Screening a collection of 178 human A/B1 extraintestinal pathogenic *E. coli* (ExPEC) strains revealed that the CVP region is highly prevalent (23%) and MLST analysis indicated that these CVP positive strains belong to several clusters and mostly to phylogroup C. The virulence of S286 is explained in part by the presence of CVP region and this region has spread in different clusters of human A/B1 ExPEC, especially in group C.

## Introduction

*Escherichia coli* is an inhabitant of the intestines of warm-blooded animals but is also a major cause of extra-intestinal diseases such as urinary tract infection, septicemia and meningitis in human or colibacillosis in poultry [1]. In pathogenic *E. coli*, different combinations of acquired virulence genes are characteristic of different pathotypes [1]. The acquisition of virulence determinants occurs preferentially in defined genetic backgrounds, which may correspond to the different major phylogenetic groups composing the species [2], [3].

Most human extra-intestinal pathogenic *E. coli* (ExPEC) strains belong to phylogenetic groups B2 and D, whereas strains belonging to groups A and B1 may be either commensal or intestinal pathogens and are rarely responsible for extraintestinal infections [3]–[7]. For instance, the prevalence of groups A and B1 is respectively 8% and 2% among neonatal bacteremia and meningitis strains [3] and in a collection of 161 *E. coli* strains responsible for bacteremia in adults, rates of groups A and B1 were 20% and 7% respectively [8]. This contrasts with the prevalence of groups A and B1 among avian pathogenic *E. coli* (APEC) strains which may reach 38–71% and 1.8–15.5% respectively, according to different studies [9]–[11]. ExPEC strains are characterized by several virulence factors, including adhesins, invasins, protectins and toxins, as well as several uptake systems for essential nutrients such as iron (iron-uptake systems) [12], [13]. Most A and B1 ExPEC strains harbor fewer virulence factors and show lower experimental virulence than strains belonging to groups B2 and D [6], [10].

Extraintestinal virulence genes are frequently clustered in chromosomal genomic regions acquired by horizontal transfer and termed pathogenicity islands, or on large plasmidic regions [14]–[16]. One such plasmidic region has been recently described in detail in several *E. coli* strains. Seven sequenced plasmids, one of human, five of avian and one from environmental origin were shown to harbor a complete or almost complete conserved region named Conserved Virulence Plasmidic (CVP) region notably containing 8 operons or genes [17]–[23]. The *iro*, *iuc*, and *sit* loci encode different iron-uptake systems, namely salmochelin, aerobactin and the *sit* system [24]–[26]. Plasmidic *Omp*T (*Omp*T_p_) encodes a putative outer membrane protease (omptin) and *cva* encodes colicinV, whereas *hly*F encodes α-haemolysin, a pore-forming toxin [27], [28]. The e*ts* operon encodes a type I secretion system and *iss* is the increased serum survival gene [18]. In human ExPEC, the prevalence of the CVP region is 10–20% of uropathogenic *E. coli* (UPEC) and 50–60% of neonatal meningitis *E. coli* (NMEC) and CVP region is found mainly in group B2 [5], [13], [19] while it is largely distributed in the 4 main phylogroups in APEC strains [11], [13], [29], [30].

Although B2/D strains constitute the majority of human ExPEC strains, a few A/B1 strains exhibit unexpected pathogenicity, exemplified by high lethality in a mouse model, similar to that obtained with B2/D strains [6]. The genetic determinants involved in this unexpected pathogenicity have not been well studied. We recently described a K1-negative *E. coli* strain, named S286, responsible for a case of neonatal meningitis and virulent in a mouse model of septicemia [31]. Triplex PCR phylogrouping [32] suggested that it belonged to phylogenetic group A while MLST analysis showed that it belongs to the phylogroup C, which is a subgroup erroneously classified as A group by PCR triplex although it is closely related to major group B1 [33], [34]. S286 was also shown to harbor a TEM-52 extended-spectrum beta-lactamase (ESBL) associated with resistance to nalidixic acid, chloramphenicol and tetracycline and, interestingly, harbored 3 different iron-uptake systems: aerobactin, salmochelin and yersiniabactin which are rarely present together in human A/B1 strains [31]. We suspected that the simultaneous presence of aerobactin and salmochelin might be related to the presence of a plasmid containing a CVP region, which may potentially explain the unexpected virulence of this C group strain.

In this study, we showed that S286 belongs to the sequence O-type ST23_O78_ and harbors a CVP region. We performed a gene expression analysis of the CVP region and investigated *in vivo* its role in the virulence of S286. To examine the potential diffusion of the CVP region among A/B1 strains, we conducted an epidemiological study of the distribution of this region in A/B1 ExPEC strains and especially in phylogroup C.

## Materials and Methods

### Bacterial strains

The neonatal meningitis strain S286 has previously been shown to belong to group A by triplex PCR, and to harbor genes encoding three iron-uptake systems – yersiniabactin, aerobactin and salmochelin [31]. Strain S286rifR is a rifampicin-resistant mutant of S286. The rifampicin resistance was obtained through mutation of S286 on selective medium containing rifampicin (250 µg/ml). Strain J53, derived from strain K-12, is resistant to rifampicin and was used for plasmid conjugal transfer as a colicin-sensitive strain [35]. The sequenced *E. coli* meningitis strain S88 (O45:K1:H7), belonging to the phylogenetic group B2, contains a virulence plasmid of 134 kb, designated pS88 harbouring the genes characteristic of the CVP region [19].

To investigate the epidemiology of the CVP region in ExPEC strains belonging to groups A and B1, we selected all the A/B1 strains (n = 178) within 8 different collections of 1945 strains responsible for meningitis, urinary tract infection and/or bacteremia in children [4], [5], [36], and for which the phylogenetic group had been determined by triplex phylogrouping PCR [32]. The 8 collections are issued from the Microbiology Unit of the Robert-Debré Hospital and from the National Reference Center of extraintestinal *E. coli*. Three collections belong to the National Reference Center of extraintestinal *E. coli* containing strains isolated from children from all over France involved in either meningitis (n = 325), urinary tract infections (n = 141) or bacteremia (n = 163). Five collections are issued from the Microbiology Unit of the Robert-Debré Hospital. Three collections related to bacteremia, in infants under 3 months (n = 98), in infants of more than 3 months with urinary tract infection (n = 16) and in children with digestive tract diseases (n = 22). The other two collections include strains from urinary tract infections without bacteremia, one collection with Extended-spectrum beta-lactamase (ESBL) producing *E. coli* (n = 82) and one with non-ESBL producing *E. coli* (n = 1098). All the collections were constituted since 2006 excepted the collection of *E. coli* meningitis of the National Reference Center of extraintestinal *E. coli* which started in 1994.

A collection of 50 *E. coli* strains of A/B1 group isolated from healthy children's feces during a routine visit to the pediatrician was also screened for CVP. Finally, a group of 9 strains belonging to the ECOR collection and representative of phylogenetic groups A and B1 were also used [37]. ESBL-producing isolates were screened by the double disk test for synergy between third-generation cephalosporins and amoxicillin-clavulanate.

### Plasmid curing and conjugual plasmid transfer

Strain S286 was grown for 18 h in Luria Bertani (LB) broth at 37°C with shaking. The culture was then diluted to 10^5^ CFU/mL, and a final concentration of 2.5% of sodium dodecyl sulfate was added, as previously described [19]. Briefly, after 18 h of growth with shaking, the cultures were plated on LB agar. After overnight incubation, 500 colonies were screened for colicin production by picking them out on LB agar plates overlaid with a suspension of strain J53 (sensitive to colicin). Test strains were assumed to have lost the plasmid containing the colicin operon if they were unable to inhibit the growth of strain J53. This was confirmed by using the CVP PCR method described below.

Conjugal plasmid transfer was performed as previously described [19]. Selective medium contained rifampicin (250 µg/mL) plus cefotaxime (1 µg/mL), or chloramphenicol (20 µg/mL) or colicin. For the last selective medium we used a medium containing 250 µg/mL rifampicin plated with our mating mixture, then we streaked it with several lanes of a mutant of S286rifR and, after 18 h of incubation at 37°C, we harvested all colonies appearing in the inhibition zone. The potential transconjugants thus obtained were checked for colicin production and the presence of the CVP genes sought by PCR.

### Detection of genetic determinants by PCR

The phylogenetic group was initially determined with a PCR-based method that detects the *chuA* and *yjaA* genes and an anonymous DNA fragment, TspE4.C2, as previously described [32]. Strains initially assigned to phylogroup A were rescreened with the new phylogroup C specific PCR as recently described [33].

We investigated the distribution of genetic determinants characteristic of CVP regions described in both human ExPEC and avian pathogenic *E. coli* (APEC) [18], [19]. The presence of *ompTp*, *etsC*, *iss*, *hlyF*, and *cvaA* together with the genes encoding salmochelin (*iroN*), aerobactin (*iucC*) and the iron-uptake system SitABCD (*sitA*), considered to be a signature of the CVP region, was sought by PCR as previously described [5], [19] along with *cia*, colicin Ia, *eitB*, ABC iron transporter and *tsh*, temperature-sensitive hemagglutinin, which are inconsistently associated with CVP. In brief, gene screening was performed by using two multiplex PCRs in a 50-µL volume with 25 µL of 2X Qiagen Multiplex PCR Master Mix (Qiagen), 5 µL of 5X Q solution, 5 µL of a primer mix (final concentrations in the hexaplex CVP PCR: 1 µM for *eitB*, *iss*, *iucC* and *sitA* and 2 µM for *cia* and *iroN*; final concentrations in the pentaplex CVP PCR: 1 µM for *cvaA*, *hlyF* and *tsh* and 2 µM for *etsC* and *ompT_p_*), 10 µL of distilled water and 5 µL of bacterial lysate, using an iCycler thermal cycler (Bio-Rad, Marnes La Coquette, France) in the following conditions: DNA denaturation and polymerase activation for 15 min at 95°C; 30 cycles of 30 s at 94°C, 90 s at 55°C, and 90 s at 72°C; and a final extension step for 10 min at 72°C. Samples were separated on 3% agarose electrophoresis gel.

Other virulence genes (*sat*, serin protease autotransporter toxin; *vat*, vacuolating autotransporter toxin; *cdt*, cytolethal distending toxin; *hek/hra*, hemagglutinin; *clbN/clbB*, colibactin, and *tcpC*, which encodes TIR domain-containing protein) were sought by PCR as described elsewhere [5], [38], [39].

PCR was performed to detect the *wzy* gene specific of the O78 O-antigen gene cluster. The primers pairs O78.1 (5′-TACGACAAGGTGTTGCTGCT-3′) and O78.2 (5′-AAATAGCAATAGGGCGGAAA-3′) generating a fragment of 184 bp were based on O78 cluster sequence (Genbank acc. Number FJ940775). Briefly, PCR was performed in a 50-µL volume with 25 µL of AmpliTaq Gold PCR Master Mix (Applied Biosystems), 5 µL of 5X Q solution, 2 µL of each primer at 10 mM, 16 µL of distilled water and 5 µL of bacterial lysate, using an iCycler thermal cycler (Bio-Rad, Marnes La Coquette, France) in the following conditions: DNA denaturation and polymerase activation for 15 min at 95°C; 30 cycles of 30 s at 94°C, 30 s at 55°C, and 30 s at 72°C; and a final extension step for 10 min at 72°C. Samples were separated on 2% agarose electrophoresis gel. ECOR 70 and ETEC H10407 [40] strains were used as positive control. Specificity of PCR was assessed using template DNAs extracted from 130 O-reference strains as previously described [41].

### Plasmid analysis

Plasmid profiling was performed using S1-nuclease digested plasmid DNA, separated by pulsed-field gel electrophoresis (PFGE) on 1% agarose gel, as previously described [42]. Subsequently, Southern blot hybridization was performed [43]. First, digested products were transferred to a Hybond-N+ membrane (Amersham, GE Healthcare Life Sciences) by capillary blotting. The probes for Southern hybridization were synthesized by PCR using the primers served for *iroN* and *etsC* gene detection. The probes were labelled with digoxigenin using the PCR DIG Probe synthesis Kit (Roche, Manheim, Germany) according to the manufacturer's instructions. The blot was hybridised, blocked and incubated with antibodies. Probes detection was performed on a PXi (Syngene) by chemiluminescence.

### Experimental model

We assessed the ability of the wild-type strain S286 and the cured strain to induce bacteremia and lethal infection in newborn rats, as previously described [44], [45]. Briefly, at 5 days of age the Sprague-Dawley newborn rats (Charles River Laboratories) were inoculated intraperitoneally with 100 µL of a normal saline suspension containing approximately 4×10^7^ CFU/mL or with serial ten-fold dilutions of this suspension. A tail incision was made 7 h and 24 h after inoculation, and 5 µL of blood was sampled for CFU counting. The virulence of S286 and its cured variant were also assessed in the adult mouse model as previously described [6], [31]. Animals were observed regularly (5 times a day) and those experiencing significant pain and distress defined by lethargy, abnormal posture or sustained chills where humanely euthanized using lethal isofluran anaesthesia. Only animals which did not appear ill were observed until 120 hours and were euthanized after this delay using lethal isofluran anaesthesia. Animal experiments and housing complied with Université Paris Diderot guidelines and were approved by the French Veterinary Services (Accreditation A 75-19-01).

### Gene expression analysis

Gene expression analysis of CVP region of S286 was performed in comparison to CVP region of pS88, as previously described [46]. Briefly, an overnight culture of strains S286 and S88 in Luria Bertani (LB) broth (Sigma) was diluted 1/100 in LB broth and grown at 37°C with agitation until optical density at 600 nm (OD_600_) reached 0.65. These cultures represented the reference conditions for this study. Strains S286 and S88 were then grown in LB broth containing the iron chelator 2,2′-dipyridyl (Sigma, Saint Quentin Fallavier, France) at a final concentration of 200 µM, as previously described [47]. RNA was extracted with the RNeasy Mini kit (QIAGEN) according to the manufacturer's instructions and isolated with the RNase-Free DNase set (QIAGEN). Quantitative reverse transcription-PCR (qRT-PCR) were performed in microplates (Eurogentec) as previously described [46]. The fold change in the abundance of the ORFs transcripts between the test condition and the reference condition was normalized on the average of 3 housekeeping genes and calculated by using the 2^−ΔΔCT^ method as previously described [46].

### Genetic diversity and phylogenetic relationships

The diversity of A/B1 group CVP+ ExPEC isolates was analysed with the semi-automated rep-PCR system (DiversiLab) as previously described [48]. Strains were also typed by MLST following the Achtmann scheme (mlst.ucc.ie/mlst/dbs/Ecoli) as previously described [5].

### Sequencing and assembly of S286

S286 was sequenced at Institut Pasteur using the Illumina high-throughput sequencing technology. Five µg of DNA was fragmented using a nebulization technique with a disposable device, according to Illumina recommendations. Generated DNA fragments, with size around 500 pb, were used for the genomic library construction, using the TruSeq DNA sample prep kit V1 (Illumina) according to the manufacturer's recommendations. The library was sequenced on Illumina HiSeq-2000 platform to produce 100 bp single reads, using TruSeq PE Cluster kit v3 and TruSeq SBS kit v3. Around 12,000,000 high-quality reads were obtained and assembled in 316 contigs using the Genomics Workbench from CLC Bio (version3). Contigs were then mapped to the pAPEC-1 plasmid (NC_0011980.1) by using BLASTn, with an E-value of 10e-5. Contigs, matching with pAPEC-1 plasmid, were then reordered using the Mummer software [49]. The identification of the gaps allowed primer design for bridging the contigs using PCR, to obtain a complete sequence corresponding to CVP region of 97,818 nt. MaGe (Magnifying Genomes) software was used for gene annotation of this sequence [50]. The DNA sequence of the CVP region has been deposited in “European Nucleotide Archive” (EMBL-EBI) with the accession number HF922624.

### Statistical analysis

Comparison of CVP prevalence between ExPEC strains and fecal carriage strains belonging to phylogroups A/B1 was based on a χ^2^ test. Comparison of bacterial counts in the rat model was based on an unpaired, two-sided Student t test. Mortality was compared with the Gehan-Breslow-Wilcoxon test implemented with GraphPad Prism 5.01 (GraphPad Software, San Diego, CA, USA). *P* values below 0.05 were considered to denote statistically significant differences.

## Results

All the genes encoding virulence factors characteristic of the CVP region (*ompTp*, *etsC*, *iss*, *hlyF*, *cvaA*, *iroN*, *iucC*, and *sitA)* and sought by PCR were present in S286, suggesting the presence of the CVP region and, possibly, of a plasmid related to sequenced plasmid pS88 [19], pAPEC-O1-ColBM [17], pAPEC-O2-ColV [18] or p-Chi7122-1 (previously named pAPEC-1) [22]. We also detected *tsh* but not *eitB* (two genes initially described in APEC-O1 and APEC-O2 strains but not in S88), while *cia*, characteristic of plasmid pS88, was not detected either. All other virulence genes sought (*sat*, *vat*, *cdt*, *clbN/clbB*, *tcpC*) were absent from S286, except *hek/hra* (Table 1).

**Table 1:** Characteristics of the S286 transconjugants and variants.

Phenotypic and genotypic characteristics	Strains					
	S286	J53	J53 pS286colV	J53 pESBL	J53 pC/TetR	S286ΔpS286colV
**Phenotypic characteristics**						
*ESBL production*	+	−	−	+	−	+
Nalidixic acid resistance	+	−	−	−	−	+
Rifampicin resistance	−	+	+	+	+	−
Tetracycline resistance	+	−	−	−	+	+
Chloramphenicol resistance	+	−	−	−	+	+
Colicin production	+	−	+	−	−	+
**Genotypic characteristics**						
*iucC*	+	−	+	−	−	−
*iroN*	+	−	+	−	−	−
*etsC*	+	−	+	−	−	−
*iss*	+	−	+	−	−	−
*ompT*	+	−	+	−	−	−
*sitA*	+	−	+	−	−	−
*hlyF*	+	−	+	−	−	−
*cvaA*	+	−	+	−	−	+
*tsh*	+	−	+	−	−	−

To determine whether the determinants of the CVP region were physically linked to a plasmid, and to analyze their relationships with the ESBL and other antibiotic resistance genes, we performed several conjugation tests between S286 and J53. We obtained 3 different transconjugants, whose characteristics are summarized in Table 1. We therefore suspected the presence of at least 3 different plasmids, one bearing the CVP region named pS286colV, one encoding the blaTEM-52 gene, and the last containing chloramphenicol and tetracycline resistance genes.

Sequencing of S286 confirmed the presence of a CVP region. A nucleotide alignment of 97 818 bp contig showed that it contains a CVP region highly homologous (98%) to that of the p-Chi7122-1 recently sequenced by Mellata *et al*. [22] meanwhile homology with those of other APEC colV-plasmids and pS88 was less marked. Regions flanking the CVP region differ from p-Chi7122-1 by only one deletion of 1981 bp (2 putative transposases) and one insertion corresponding to an ORF of 1959 bp encoding a parB-like partition protein. Moreover, the O-Antigen gene cluster of S286 could be identified and was identical to that of O78 reference strain [51].

**Figure 1:**
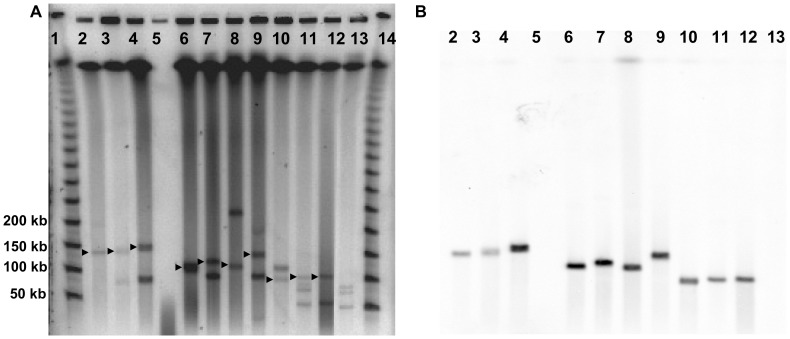
Pulsed-field gel electrophoresis of S1 nuclease-digested DNA (A) and Southern blot hybridization (B). (1) and (14) Size marker; (2) Strain 25153; (3) Strain 23791; (4) Strain 23670; (5) Strain BSE40; (6) Strain 25985; (7) Strain 30091; (8) Strain S280, (9) Strain 29597, (10) Strain PP59; (11) Strain S286; (12) Strain J53pS286colV; (13) S286ΔpS286colV. Arrows indicate plasmid bands hybridizing with *iroN* and *etsC* probes. (Only Southern blot with *iroN* probe is showed).

We cured the plasmid containing the CVP region from S286 in order to explore its role in the experimental virulence of the strain. Curing was performed by exposure to sodium dodecyl sulphate and screening of the colicin production phenotype. All 500 colonies obtained after SDS subcultures were colicin+ and thus considered as CVP+. Puzzled by the results of this method, which proved to be highly efficient with S88 [19], we screened these colonies by PCR and, surprisingly, found that 2 colonies had lost all the genes of the CVP region except for *cvaA* (Table 1). This confirmed that S286 harbours a plasmid containing a CVP region and suggested that S286 harbored two copies of the colicin V operon, one on the plasmid and a probable chromosomal copy. This was confirmed by analysis of S286 sequences (data not shown). We then analyzed the plasmidic content of S286, J53pS286colV and S286ΔpS286colV. Plasmid extraction was performed with S1-nuclease digestion (Figure 1A). Southern blot with two probes generated by PCR amplification of two characteristic genes of the CVP region, *iroN* and *etsC*, was used to confirme the presence or the absence of both genes on the same plasmid (Figure 1B). In S286, we identified 4 bands corresponding to 4 plasmids with molecular weights ranging from 50 to more than 100 kbp and the largest plasmid (∼100 kbp) carried the genes *iroN* and *etsC*. This plasmid was also present in the transconjugant J53pS286colV, but a second band of ∼50 kb was seen in this transconjuguant, suggesting that 2 different plasmids were transferred by conjugation. Unfortunately, we were unable to obtain a transconjugant bearing only the plasmid harboring the CVP region. In the cured strain, S286ΔpS286colV, we confirmed the loss of the largest plasmid.

**Figure 2:**
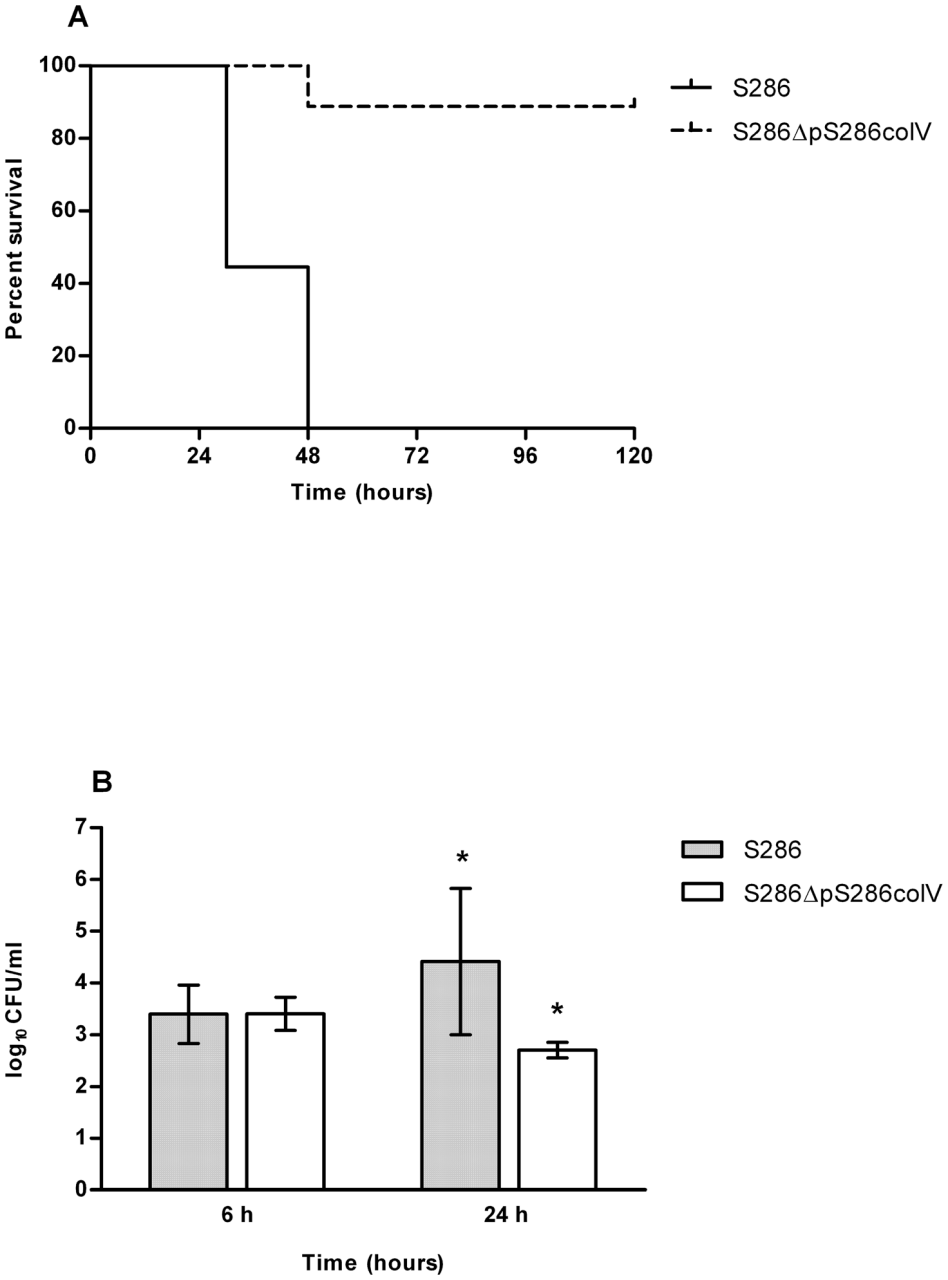
Mortality (A) and Bacteremia (B) of S286 and S286ΔpS286colV in a neonatal rat sepsis model. The same inoculum (4×10^5^ CFU) was injected intraperitoneally. *P* values below 0.05 were considered statistically significant and were denoted by *.

To assess the role of pS286colV in S286 virulence, we first used a neonatal rat sepsis model. We chose to use an inoculum of 4×10^5^ CFU as it was the smallest inoculum yielding 100% mortality with S286 after 48 h (data not shown). Ten rats were inoculated per group. ΔpS286colV was significantly less virulent than the wildtype strain, with a level of bacteremia almost 2 log CFU/mL lower at 24 h (4.41+/−1.41 vs 2.60+/−0.16 log_10_ CFU/ml, p = 0.001). Mortality was also significantly lower with S286ΔpS286colV than with S286 (20% vs 100%, p = 0.0002) (Figure 2). In contrast, in the standardized adult mouse model, all 10 animals infected with either S286 or S286ΔpS286colV died within 48 hours.

**Figure 3:**
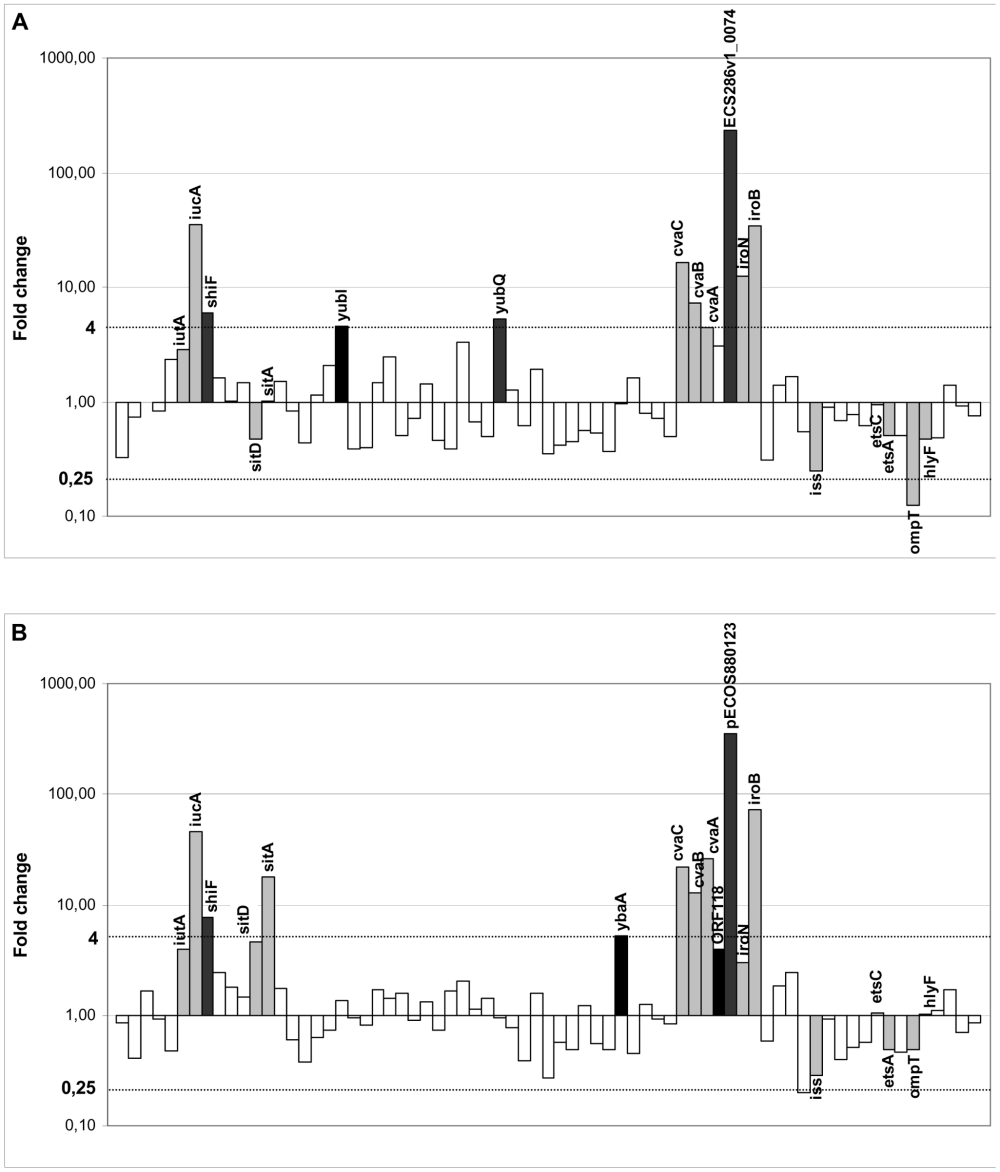
Fold changes of RNA transcripts of pS286colV (A) and pS88 (B) in iron deficiency environment. Relative expression of transcripts were calculated between growth in LB with an iron chelator comparatively to growth in LB. Only genes characteristics of the Conserved Virulence Plasmidic (CVP) region (grey) and other genes upregulated (black) are named. Fold changes were calculated by 2^−ΔΔCt^ method.

To gain insight into CVP region function of pS286colV, we performed a gene expression analysis of this region in comparison to that of the previously investigated pS88 [46]. Quantification of transcripts was obtained following growth in LB and in LB with an iron chelator to mimic the situation of iron deficiency such as that is encountered during growth in serum or urine. A total of 67 plasmid transcripts common to both strains could be retained for investigation. The relative expression of transcripts of the CVP region of each plasmid is shown in Figure 3. Overall, a similar profile of genes expression was observed between both plasmids. Remarkably we noted that the most induced transcript in both plasmids were the pECOS880123 and its orthologue EcS286v1_0074 on pS286 of yet unknown function. The only major difference was the absence of transcriptional induction of the sit operon in pS286colV.

In order to determine the prevalence, genetic diversity and phylogenetic relationships of CVP+ strains in human ExPEC A/B1 strains, we examined 178 A/B1 strains previously partially characterized. Forty-three strains were known to harbor salmochelin (*iroN*) and aerobactin (*iucC*) genes and 41 (95%) of them were found to be positive for the CVP region. Thus, the simultaneous presence of salmochelin and aerobactin in A/B1 ExPEC is almost always associated with the presence of the CVP region whose prevalence could be estimated to 23% (41/178) in A/B1 ExPEC strains. On the other hand, only 2 of the 50 *E. coli* isolated from healthy children's feces and belonging to phylogroups A/B1 harbored the 8 genes characteristic of the CVP region. Thus, CVP was far less prevalent in healthy children's fecal isolates than in ExPEC strains [23% (41/178) vs 4% (2/50), *P* = 0.002] suggesting its implication in the virulence of non B2/D ExPEC strains.

**Figure 4:**
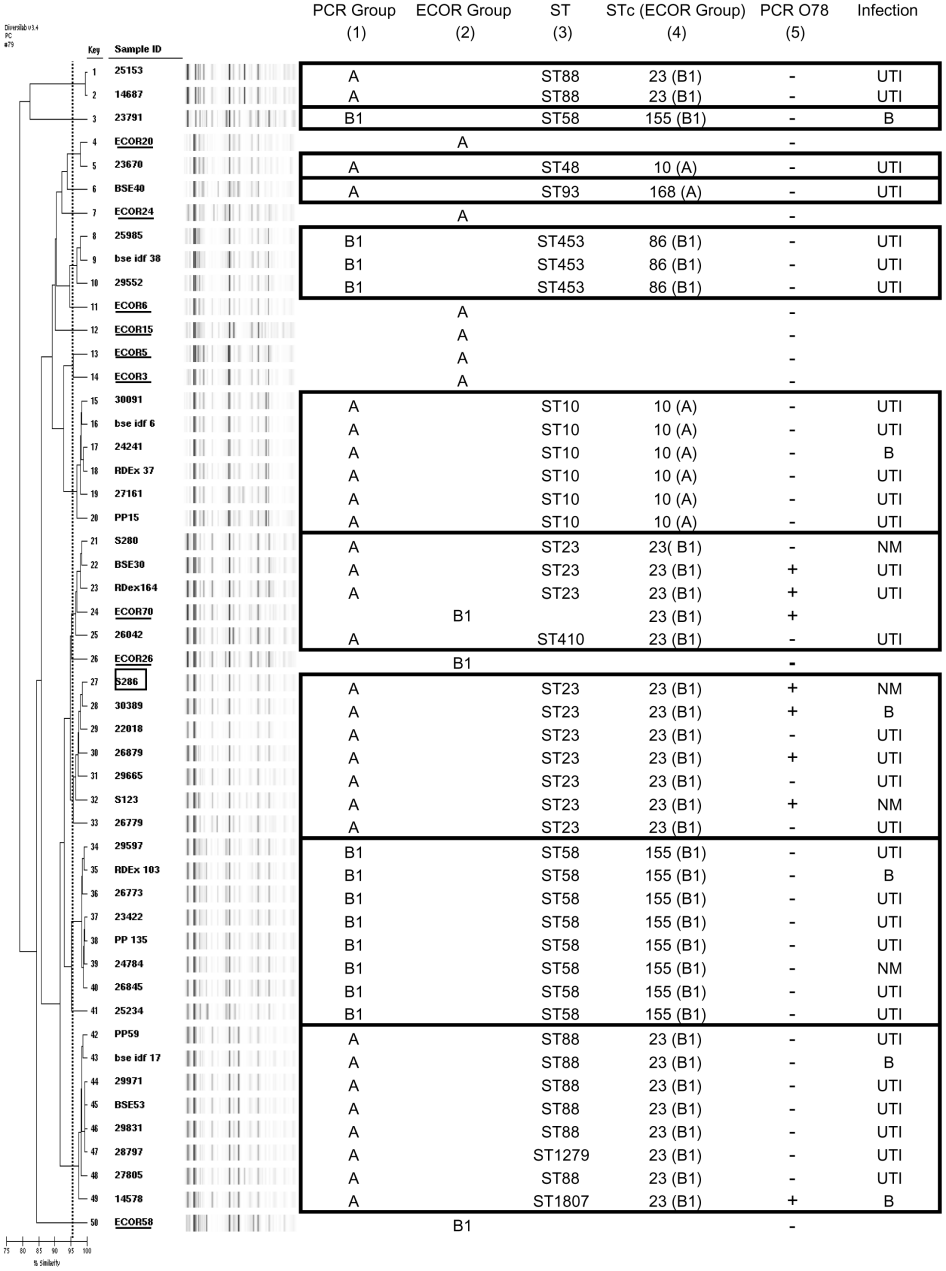
RepPCR (Diversilab®) and UPGMA dendrogram obtained with 41 A/B1 CVP+ strains and 9 ECOR strains. (1) Phylogenetic group, as determined by PCR; (2) Phylogenetic group of ECOR strains; (3) ST: sequence type according to Achtmann's scheme; (4) STc: sequence type complexes according to Achtmann's scheme (corresponding phylogenetic group in brackets); (5) PCR O78. Strains were responsible for Urinary Tract nfection (UTI), Neonatal Meningitis (NM) or Bacteremia (B). Clusters of clinical isolates are framed in black and ECOR strains are underlined.

Diversity and phylogenetic relationships of the 41 CVP+ ExPEC strains were then explored. We first used the semi-automated rep-PCR method. The dendrogram thus obtained showed 10 clusters, indicating that the CVP region is not restricted to the S286 cluster (Figure 4). As it has been previously described that PCR phylogrouping method had erroneously classified S286 in group A [31], we genotyped by MLST all of our A/B1 CVP+ExPEC strains. These latter were distributed among 10 STs and MLST analysis confirm the missclassification of numerous strains by PCR phylogrouping method. All 21 strains belonging to STc23 (containing ST23, ST88, ST410, ST1279 and ST1807), including S286, were erroneously classified as group A by PCR (Figure 4). Indeed, all these strains were confirmed to belong to the phylogroup C by the new specific PCR recently described [33] (data not shown). Finally PCR specific of O78 antigen allowed to identify 6 other strains belonging to the sequence-O-type ST_c_23_O78_ (Figure 4).

To determine whether the CVP region was actually located on a plasmid, one strain was chosen for each cluster (ten strains) and plasmid content was determined by extraction using S1-nuclease digestion and PFGE separation. Excepted the strain BSE40 which could not be characterized because of autodigestion, we showed that each strain contained from one to four plasmids with a size ranging from ∼50 to ∼250 kbp (Figure 1A). Southern blot using two probes, *iroN* and *etsC*, revealed, in each strain, the colocalization of these two genes on a unique plasmid with a size ranging from ∼50 to ∼150 kbp (Figure 1B).

## Discussion

In 2009, we described an ESBL-producing *E. coli* strain, S286, isolated from cerebrospinal fluid of a newborn, and initially shown to belong to group A by triplex PCR. Intriguingly, S286 does not harbor the K1 capsular antigen, a key virulence factor present in approximately 90% of meningitis strains [4], but it does harbor 2 iron-uptake systems (salmochelin and aerobactin), which are rarely both present in human A/B1 strains. Here we show that the aerobactin and salmochelin genes are present on a plasmid, together with several genes characteristic of the CVP region, initially described in human and avian B2 ExPEC strains [18], [19]. Sequencing of CVP region of pS286colV showed a high homology with the CVP region of the recently sequenced largest plasmid of O78:H9 APEC Chi7122 (pChi7122-1, previously named pAPEC-1) [22]. To our knowledge this is only the second CVP region sequenced from a human ExPEC meanwhile similar regions have been sequenced from five APEC strains. Phylogenetic analysis of colV plasmids sequences had suggested that these plasmids fell into two lineages [21]. The human colV plasmid sequenced (pS88) was found to belong to the first lineage [21]. The strong homology between both CVP regions of pS286colV and pChi7122-1 indicates that the second sequenced human colV plasmid belongs to the second lineage. Therefore the two lineages of plasmids bearing CVP region appear to have a zoonotic potential.

We have shown previously that the plasmid containing the CVP region plays a key role in the virulence of the meningitis strain S88, yielding a level of bacteremia more than 2 log higher than the cured strain in a neonatal rat sepsis model [19]. S286 appears to be less virulent than S88, as an inoculum of 4×10^5^ CFU was necessary to induce a high mortality rate, compared to less than 10^3^ CFU for S88, as previously reported [3]. However, we showed that a cured variant induced a lower level of bacteremia and a lower mortality rate than the wild type strain, thus indicating that the plasmid bearing CVP plays an important role in the virulence of S286. Dozois *et al*. were the first authors who assessed the virulence of the plasmid pChi7122-1, especially the role of aerobactin and *iro* locus [52], [53]. More recently, Mellata *et al*. experiments showed that the plasmid pChi7122-1 plays a role in dissemination of bacteria in blood and internal organs of infected chickens [54]. Importantly, we showed that the plasmid does not explain by itself the pathogenicity of S286. Indeed, in the adult mouse model, S286ΔpS286colV was as virulent as S286 meaning that the chromosome of S286 or another plasmid could also carry virulence genes. However, the difference observed between the adult model and the neonatal rat model could be in part explained by the impaired innate defense of the neonatal rats, notably, as previously described, due to deficit in complement components as human neonates [55], [56].

Gene expression analysis of pS286 in an iron deficient environment appears very close to that of pS88 confirming the most significant results obtained with the B2 meningitis strain S88. Indeed, the high induction of two ORFs encoding proteins of unknown function (pECOS880123 and *shiF*) was very similar in both strains; as well as transcript production of operons encoding siderophores. We also confirm the absence of induction of several genes with putative role in virulence such as *iss*, *hlyF*, *ompT_p_*, *mig14* and *eit*. Although all these results suggest that CVP region may be expressed similarly in two very different genetic backgrounds (group C versus group B2), one major discrepancy was observed with the sit operon. Indeed, *sitA* and *sitD* were not upregulated in our study in contrast to previous transcriptional analyses in urine or serum showing a strong upregulation [57], [58] although the *sit* operon in pS286colV differs from that of pS88 by only 2 single nucleotide polymorphisms which do not affect the promoter. However,

As S286 belongs to phylogroup C, closely related to major group B1, we examined the prevalence of CVP among a large collection of human ExPEC strains initially classified as A/B1. Although the prevalence of several virulence genes belonging to CVP region among major phylogenetic groups has been previously described in human ExPEC and APEC [5], [10], [13], [19], [21], [59]–[61], the distribution of gene combinations characteristic of CVP region among phylogenetic subgroups and more specifically among STs has been seldom performed [5], [19]. The rate of CVP+A/B1 ExPEC strains was surprisingly high, close to 23%. Analysis of genetic relatedness by repPCR showed that this high prevalence of CVP was not related to expansion of a few of clones but was associated to at least 10 clusters in our collections. MLST analysis revealed that 21/42 (50%) of the isolates belonged to the STc23 which can be associated with Group C. Altogether these results indicated that group C may constitute a reservoir of CVP region-associated plasmids among non B2/D strains. Furthermore, in our collection of A/B1 CVP+ExPEC, we found a significant percentage of O78 strains (7/42, 17%) and all O78 strains belonged to the ST_c_23 (group C).

Recently, APEC isolates have been compared to ExPEC isolates from UTIs and neonatal meningitis, revealing some similarities in serogroups, phylogenetic groups, virulence genotypes, and abilities to cause disease in certain animal models [10], [11], [13], [62], [63]. Of note, whereas the clonal groups O1:K1, O2:K1 or O18:K1 are well represented in human and avian ExPEC [64], [65], serogroup O78 has rarely been described in human ExPEC studies describing O serogroupes [10], [11], [62], [66] and the lower zoonotic risk of O78 versus O1:K1, O2:K1 and O18:K1 remains to be explained. However, an outbreak of urinary tract infections caused by an enteroaggregative *E. coli* O78:H10 has been recently described [67].

To conclude, we show that the paradoxical virulence of S286, which does not belong to B2/D group, is in part related to the presence of a CVP region. Moreover, this CVP region is also largely prevalent in human “commensal” phylogroups, and distributed among different clusters, especially group C. Therefore, the CVP region may partly explain the unexpected virulence of some A/B1 and notably group C strains. However, further studies are needed to search for other genetic determinants that could fully support the virulence of S286 and by extension of phylogroup C.
